# Transcriptional Profiling of Primordial Germ Cells During Chicken Embryonic Development

**DOI:** 10.3390/vetsci13070662

**Published:** 2026-07-07

**Authors:** Mingyang Jin, Jingkang Huang, Chao Qin, Kaixuan Yang, Fuquan Xiao, He Meng

**Affiliations:** 1 Shanghai Key Laboratory of Veterinary Biotechnology, Department of Animal Science, School of Agriculture and Biology, Shanghai Jiao Tong University, Shanghai 200240, China; jmyparadise@sjtu.edu.cn (M.J.); huangjingkang@sjtu.edu.cn (J.H.); qin_chao@sjtu.edu.cn (C.Q.); xiaofuquan@sjtu.edu.cn (F.X.); 2 Animal Husbandry and Veterinary Research Institute, Shanghai Academy of Agricultural Science, Shanghai 200030, China; yangkaixuan007@hotmail.com

**Keywords:** chicken, primordial germ cells, transcriptional profile, germ cell differentiation, genetic resource conservation, genome editing

## Abstract

Primordial germ cells (PGCs) are the earliest precursors of gametes and essential cellular materials for poultry germplasm conservation and genetic modification. In this study, PGCs isolated from 2.5-day male and female Silkie chicken embryos were defined as circulating PGCs (cPGC_M and cPGC_F), whereas PGCs isolated from 8.5-day male and female embryos were defined as gonad-derived PGCs (gPGC_M and gPGC_F). RNA sequencing with three biological replicates per group was performed to characterize developmental-stage- and sex-associated transcriptional programs. Comparative transcriptomic analyses identified stage-associated differences in pathways related to cell–matrix interaction, focal adhesion, PI3K–Akt signaling, and stem cell pluripotency, with more differentially expressed genes detected in the female than in the male stage comparison. Sex-biased expression was detectable at the circulating stage, mainly reflecting W- and Z-linked expression differences, and the number of sex-biased genes was greater after gonadal colonization, primarily because more autosomal differentially expressed genes were detected. Candidate genes and pathways, including *HINTW*, *DMRT1*, *SOX2*, *EDNRB*, *LPAR4*, *GFRA3*, ECM–receptor interaction, ribosome-related modules, and calcium signaling, were associated with these comparisons and are presented as targets for future validation rather than as confirmed regulators. Because the study used three biological replicates per group, RT-qPCR corroboration was limited to six female stage-associated genes, and no functional perturbation assays were performed, the findings are descriptive and do not establish causal mechanisms of PGC migration, gonadal colonization, or sex differentiation. Within these limitations, the dataset provides an exploratory resource for subsequent studies of chicken PGC biology and biotechnology.

## Simple Summary

Primordial germ cells (PGCs) are the founder cells of gametes and are important cellular materials for poultry genetic resource conservation and genome editing. In chickens, PGCs can be collected during migration in the embryonic circulation and after colonization in the gonads, but how their transcriptional programs differ between these developmental stages and between sexes remains incompletely understood. This study used RNA sequencing to compare male and female Silkie chicken PGCs at 2.5 and 8.5 days of incubation. The analysis focused on developmental-time changes and sex-associated transcriptional differences, supported by differential expression analysis, GO and KEGG enrichment, gene set enrichment analysis, and expression-trend clustering. The work provides an exploratory transcriptomic description of developmental-stage- and sex-associated expression differences in chicken PGCs and identifies candidate genes and pathways for future functional investigation. Because the study included three biological replicates per group and did not include functional perturbation experiments, the findings should be interpreted as hypothesis-generating rather than as evidence of causal mechanisms.

## 1. Introduction

Primordial germ cells (PGCs) are the precursors of gametes and the earliest cells specified to the germline fate during embryonic development. They ultimately differentiate into spermatozoa or oocytes, forming the cellular basis for the intergenerational transmission of genetic and epigenetic information [1]. In mammals, PGCs are generally specified from the epiblast of the early post-implantation embryo. Their specification depends on BMP and WNT signaling and key transcriptional regulators, including BLIMP1 (PRDM1), PRDM14, and AP-2γ (TFAP2C), and is accompanied by the reactivation of pluripotency-associated genes and extensive epigenetic reprogramming [2,3]. After specification, PGCs migrate to and colonize the developing gonads, where somatic gonadal cells, local hormones, the extracellular matrix (ECM), and sex-specific signals direct their differentiation into oogonia or prospermatogonia [4]. Thus, PGCs are essential for germline development, species reproduction, genetic inheritance, and germplasm conservation.

Compared with mammals, birds differ markedly in egg structure, PGC migration, and genetic manipulation strategies. Mammalian fertilized eggs are single-cell embryos and can be genetically manipulated through pronuclear or cytoplasmic microinjection and nuclear transfer. In contrast, avian eggs contain abundant yolk and albumen, and the embryo has already developed into a blastoderm of approximately 55,000 cells at oviposition. This structure makes single-cell microinjection impractical. Consequently, poultry genome editing and transgenic breeding generally require germline manipulation using PGCs as cellular vectors.

A major distinction between avian and mammalian PGCs is their migration route. Mammalian PGCs migrate interstitially through the yolk sac, hindgut, dorsal mesentery, and genital ridge, guided mainly by chemotactic signaling and somatic cell cues. In birds, early PGCs enter the vascular system, particularly the dorsal aorta, and are transported through the bloodstream before leaving the vasculature around Hamburger–Hamilton stage 17 and migrating toward the gonadal ridges [5,6]. This blood-borne migration provides a valuable window for avian genetic manipulation. PGCs can be collected from embryonic blood at 2–3 days of incubation, expanded and genetically modified in vitro, and subsequently injected into recipient embryos to produce germline chimeras and gene-edited offspring [7,8]. Furthermore, semen cryopreservation cannot preserve maternal genetic information, including the W chromosome and mitochondrial DNA, while avian embryo cryopreservation remains technically difficult because of the high yolk content. PGC cryobanks therefore provide an effective strategy for preserving both paternal and maternal genetic information [9,10]. Accordingly, PGCs are important for poultry genome editing, transgenic breeding, germline chimera production, and the preservation of endangered poultry genetic resources.

During chicken embryonic development, PGCs can be obtained at two key stages. At 2–3 days of incubation (HH14–17), circulating PGCs can be collected from the dorsal aortic blood. At 7–9 days, after gonadal colonization, PGCs can be isolated from the embryonic gonads. At 2.5 days, PGCs are actively circulating, migrating, and homing toward the gonads while maintaining germ cell identity and migratory competence. By 8.5 days, they have largely completed gonadal colonization and are increasingly regulated by gonadal somatic cells, the ECM, local signaling molecules, and sex-associated microenvironmental cues. Therefore, the difference between 2.5 and 8.5 days represents both a developmental transition and a spatial shift from the bloodstream to the gonadal microenvironment.

However, systematic information remains limited regarding the transcriptional remodeling of chicken PGCs during this transition. It remains unclear whether developmental stage is the primary source of transcriptomic variation, whether male and female PGCs already exhibit molecular differences during circulation, and whether these differences are enhanced or attenuated after gonadal colonization. Identifying the functional modules reorganized during this period is important for understanding early chicken germ cell development and for optimizing sampling time, culture systems, and recipient matching in PGC-based genome editing and genetic resource conservation.

To address these questions, this study examined male and female PGCs from Silkie chicken embryos at 2.5 and 8.5 days of incubation. RNA sequencing was combined with DESeq2-based differential expression analysis, GO and KEGG enrichment analysis, gene set enrichment analysis (GSEA), and expression-trend clustering to compare transcriptional differences across developmental stages and between sexes. The study aimed to characterize the transcriptional transition from blood-borne migration to gonadal colonization, evaluate developmental-stage- and sex-associated expression patterns, and identify candidate genes and pathways for subsequent functional investigation. As an exploratory transcriptomic study, it did not test the causal roles of individual genes or pathways in migration, colonization, or sex differentiation. These findings provide a transcriptomic basis for future studies of chicken PGC culture, genome editing, and genetic resource conservation.

## 2. Materials and Methods

### 2.1. Experimental Materials and Embryo Incubation

Fertilized Silkie chicken (*Gallus gallus domesticus*) eggs used in this study were provided by the Animal Husbandry and Veterinary Research Institute, Shanghai Academy of Agricultural Science, Shanghai, China. Before incubation, eggshell surfaces were disinfected by wiping them with cotton soaked in 75% ethanol (Yongxing Incubation Equipment, China). Eggs were incubated in a constant-temperature and constant-humidity incubator at 37.8 °C and 55–65% relative humidity and were automatically turned every 2 h. Samples were collected at 2.5 days of incubation (60–65 h; HH14–17) and at 8.0–8.5 days of incubation. The former were used to obtain circulating PGCs (cPGCs), and the latter were used to obtain gonad-derived PGCs (gPGCs). The materials used in this study were fertilized Silkie chicken eggs and embryonic samples from a formal breeding unit. Sample collection, embryo manipulation, and cell culture were performed in accordance with laboratory biosafety management regulations and relevant animal welfare requirements. All animal experiments were reviewed and approved by the Animal Ethics Committee of Shanghai Jiao Tong University. The approval number was 202504005, and the approval date was 22 April 2025.

### 2.2. Experimental Grouping

According to embryonic developmental time and sex, samples were divided into four groups: 2.5-day female PGCs (cPGC_F), 2.5-day male PGCs (cPGC_M), 8.5-day female PGCs (gPGC_F), and 8.5-day male PGCs (gPGC_M). cPGCs were derived from the dorsal aortic blood of 2.5-day embryos, whereas gPGCs were derived from the gonads of 8.5-day embryos. Three biological replicates were included for each group, yielding a total of 12 RNA-seq libraries. Each biological replicate was obtained from an independent embryo to ensure independence among replicates. Because each group contained only three biological replicates, the study was intended to identify reproducible group-level expression patterns rather than to provide a precise population-level estimate of inter-individual variability.

### 2.3. Embryonic Sex Identification

Genomic DNA was extracted from embryonic allantoic membrane or embryonic tissue fragments. The sex of each embryo was determined by PCR using *CHD1* gene-specific primers (forward primer: 5′-GTTACTGATTCGTCTACGAGA-3′; reverse primer: 5′-ATTGAAATGATCCAGTGCTTG-3′). PCR products were separated by 2% agarose gel electrophoresis. The Z-chromosome band was approximately 600 bp, and the W-chromosome band was approximately 450 bp. Male embryos (ZZ) showed a single 600 bp band, whereas female embryos (ZW) showed both 600 bp and 450 bp bands; sex was determined accordingly.

### 2.4. Isolation and Culture of PGCs

#### 2.4.1. Collection of PGCs from Embryonic Blood at 2.5 Days of Incubation

Embryos incubated for 60–65 h were opened at the blunt end with an approximately 1 cm window. Under a stereomicroscope, a microinjection needle was inserted into the dorsal aorta with an aspiration pressure of 10–15 kPa, and 5–10 µL of blood was collected and transferred into a centrifuge tube containing 100 µL of complete PGC culture medium. Samples were centrifuged at 2500 r/min for 4 min, the supernatant was discarded, and the cells were resuspended in complete PGC culture medium and seeded into 48-well plates (250 µL per well). Cells were cultured at 37.5 °C in a 5% CO_2_ incubator, and the medium was replaced every 2 days. Each embryo sample corresponded independently to one centrifuge tube and one culture well to maintain the independence of biological replicates.

#### 2.4.2. Collection of PGCs from Embryonic Gonads at 8.5 Days of Incubation

Embryos incubated for 8.0–8.5 days were dissected under a stereomicroscope to expose the abdominal cavity. Bilateral gonads in male embryos or the developed left gonad in female embryos were isolated and placed in centrifuge tubes containing complete PGC culture medium. StemPro Accutase was added, and the tissues were digested at 37 °C for 20 min. Cells were collected by centrifugation at 2500 r/min, resuspended in complete PGC culture medium, and seeded into 48-well plates for culture.

#### 2.4.3. Complete PGC Culture Medium

Complete PGC culture medium was prepared according to Altgilbers et al. [11], and the defined avian PGC culture system described by Whyte et al. [12] with avian KO-DMEM as the basal medium. The medium was sterilized by filtration through a 0.22 µm membrane, aliquoted, stored at 4 °C, and prewarmed to 37 °C before use.

#### 2.4.4. Culture Establishment, Expansion, and Selection of Samples for RNA-Seq

PGC cultures were initiated in parallel from a total of 150 fertilized Silkie chicken eggs. Cultures derived from embryonic blood and embryonic gonads were maintained using the same complete PGC culture medium and incubation conditions. The complete medium contained factors intended to support PGC proliferation and maintenance while limiting spontaneous differentiation. Because blood-derived and gonad-derived cultures expanded at different rates, the culture duration and passage number were not prospectively standardized; instead, the harvest criterion was based on viable cell number and cell viability. Cells were counted during culture and were harvested when the viable cell number first reached 1 × 10^7^ and cell viability exceeded 90%. Twelve independent embryo-derived cultures showing typical PGC morphology and satisfactory expansion, comprising three biological replicates for each of the four experimental groups, were selected for RNA extraction and sequencing. The initial number of isolated PGCs, the number of cultures successfully established, and the overall isolation/culture establishment efficiency were not systematically recorded.

### 2.5. Identification of PGCs

Cultured PGCs were identified by (1) morphological observation, in which the cells appeared round or oval, were highly refractile, and formed morula-like clusters; and (2) indirect immunofluorescence staining for SSEA-1. DiI labeling and DAPI nuclear counterstaining were used to assess signal colocalization in merged images. The cultures selected for sequencing were visually dominated by cells with PGC-like morphology in the examined fields. However, cell population purity was not quantitatively assessed by flow cytometry or systematic marker-positive cell counting, and negative markers for hematopoietic, endothelial, or gonadal somatic cells were not examined. Morphological observation and SSEA-1 immunofluorescence were therefore used for qualitative phenotypic identification rather than for quantitative purity determination.

### 2.6. RNA Extraction and Quality Assessment

PGC samples from the four groups were collected, and total RNA was extracted using the FastPure Cell/Tissue Total RNA Isolation Kit V2 (RC112-01; Vazyme, Nanjing, China). RNA concentration and purity were assessed using a NanoDrop 2000 spectrophotometer (Thermo Fisher Scientific, Waltham, MA, USA), with A260/A280 ≥ 1.8 and A260/A230 ≥ 2.0 as quality thresholds. RNA integrity was evaluated using an Agilent 2100 Bioanalyzer (Agilent Technologies, Santa Clara, CA, USA), and samples with RIN ≥ 8.0 were used for subsequent library construction. The RNA-seq samples were cultured rather than freshly isolated PGCs. For each selected culture, cells were harvested for RNA extraction when the viable cell number first reached 1 × 10^7^ and cell viability exceeded 90%.

### 2.7. Library Construction and Transcriptome Sequencing

Qualified RNA samples were used to construct strand-specific libraries with the VAHTS Universal V6 RNA-seq Library Prep Kit (Vazyme, China). Paired-end 150 bp sequencing was performed on an Illumina NovaSeq 6000 platform, with a target output of no less than 6 Gb of clean data per sample. Raw data were quality-controlled using Trimmomatic (v0.39) [13]. Clean reads were aligned to the chicken reference genome (GRCg7b) using HISAT2 (v2.2.1) [14]. Gene-level raw counts were generated using featureCounts (v2.0.2) [15] and used for DESeq2 analysis, whereas FPKM values were calculated for expression visualization and descriptive comparisons.

### 2.8. Differential Gene Expression Analysis

Differential expression analysis among the four comparison groups was performed using the raw gene count matrix in DESeq2 (v1.38.0) [16]. *p* values were adjusted for multiple testing using the Benjamini–Hochberg procedure. High-confidence DEGs were defined by an adjusted *p* value (padj) < 0.05 and |log2(Fold Change)| ≥ 1. The log2(Fold Change) value was used as the gene-level effect-size measure, whereas the adjusted *p* value was used to quantify statistical evidence. For each comparison written as A vs. B, A was explicitly set as the reference group, and the DESeq2 contrast was specified as contrast = c(“group”, B, A). Therefore, log2(Fold Change) was calculated as log2(B/A) from the model-fitted normalized mean expression values. Positive values indicate higher expression in the second-listed group (B), whereas negative values indicate higher expression in the first-listed group (A). Specifically, positive values indicate higher expression in gPGC_M, gPGC_F, cPGC_F, and gPGC_F for the comparisons cPGC_M vs. gPGC_M, cPGC_F vs. gPGC_F, cPGC_M vs. cPGC_F, and gPGC_M vs. gPGC_F, respectively.

### 2.9. GO, KEGG, and GSEA Enrichment Analyses

The R package clusterProfiler (v4.6.0) [17] was used for Gene Ontology (GO) [18] and KEGG [19] enrichment analyses of DEGs, covering biological process (BP), cellular component (CC), and molecular function (MF) categories, with emphasis on pathways related to PGC migration, colonization, and sex differentiation. Gene set enrichment analysis (GSEA) was performed by ranking all genes according to log2(Fold Change) values to identify directional shifts in relevant gene sets in each comparison. A positive normalized enrichment score (NES) indicated enrichment toward the latter group, whereas a negative NES indicated enrichment toward the former group [20]. For GO, KEGG, and GSEA, Benjamini–Hochberg-adjusted *p* < 0.05 was considered statistically significant. Expression-trend clustering was visualized using pheatmap (v1.0.12) [21], and KEGG enrichment analysis was used to characterize the functional features of each trend module.

### 2.10. Targeted RT-qPCR Corroboration

To provide targeted corroboration of selected RNA-seq expression trends in the female developmental-stage comparison, total RNA from three independent biological replicates of cPGC_F and gPGC_F was reverse-transcribed using a commercial cDNA synthesis kit according to the manufacturer’s instructions. RT-qPCR was performed using an SYBR Green-based assay for *AXIN2*, *DACT2*, *EDNRB*, *LPAR4*, *TLN1*, and *SOX2*. GAPDH was used as the reference gene. Relative expression was calculated using the 2^−ΔΔCt^ method, with cPGC_F as the calibrator. RT-qPCR was not performed for male samples or for representative Z- and W-chromosome genes; therefore, the male developmental-stage and sex-biased comparisons were evaluated by RNA-seq only. Female samples were selected for targeted RT-qPCR because the female developmental-stage comparison was the principal focus of this validation and showed a substantially larger number of DEGs than the corresponding male comparison (385 versus 23).

### 2.11. Statistical Analysis

All analyses were performed in the R environment (v4.2.0). PCA plots, volcano plots, and sample-distance heatmaps were generated using ggplot2 (v3.4.0) [22]. DEG heatmaps were drawn using pheatmap (v1.0.12), and GO/KEGG enrichment bubble plots were generated from clusterProfiler outputs. For transcriptomic differential-expression and enrichment analyses, statistical significance was defined as Benjamini–Hochberg-adjusted *p* < 0.05. RT-qPCR data are presented as the mean ± standard error of the mean (SEM) from three biological replicates (*n* = 3), and groups were compared using a two-tailed unpaired Student’s *t*-test; *p* < 0.05 was considered statistically significant. Within-group inter-individual variability was assessed descriptively from the dispersion of biological replicates in PCA and from sample-level expression distributions. Because each group contained only three biological replicates, no formal population-level variance-component analysis was performed.

### 2.12. Use of Generative Artificial Intelligence

During the preparation of this manuscript, the authors used ChatGPT (OpenAI, GPT-5.5) solely to assist with English-language editing and to improve the clarity, grammar, and organization of the text. The tool was not used to generate research data or graphics, perform statistical analyses, or determine the scientific interpretation or conclusions. All AI-assisted output was critically reviewed and edited by the authors, who take full responsibility for the content of this publication.

## 3. Results

### 3.1. Isolation, Culture, and Identification of Silkie Chicken PGCs at the E2.5 Circulating Stage and E8.5 Gonadal Colonization Stage

**Figure 1:**
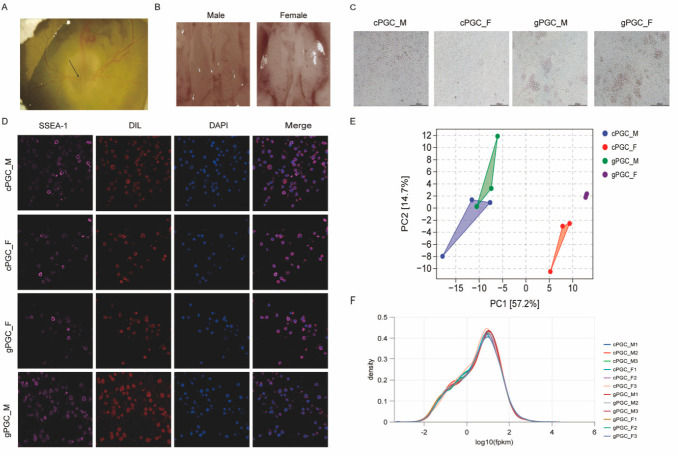
Isolation, culture, and immunofluorescence identification of PGCs. (**A**) Representative images of chicken embryos at the indicated developmental stages; arrows indicate the vascular/blood region used for the isolation of circulating PGCs. (**B**) Representative morphological images of male and female embryonic gonads used for the isolation of gonad-derived PGCs. (**C**) Morphological observation of the four cultured PGC groups, including male circulating PGCs (cPGC_M), female circulating PGCs (cPGC_F), male gonad-derived PGCs (gPGC_M), and female gonad-derived PGCs (gPGC_F). (**D**) Immunofluorescence staining of the four PGC groups; SSEA-1 fluorescence signals colocalized with DiI labeling and DAPI nuclear staining in merged images, indicating that the isolated and cultured cells exhibited PGC-associated phenotypic characteristics. (**E**) Principal component analysis (PCA) of RNA-seq samples from male and female circulating PGCs and gonadal PGCs. (**F**) Density distribution of log10(FPKM) values across all RNA-seq samples. In panels (**E**) and (**F**), different colors represent the four PGC groups, as indicated in the legends.

To obtain transcriptomic samples of Silkie chicken primordial germ cells (PGCs) at distinct developmental stages and from both sexes, PGCs were isolated from the embryonic blood of E2.5 embryos at Hamburger–Hamilton stages 14–17 (HH14–17) and from the gonads of E8.5 embryos at HH34–35 (Figure 1A,B). Morphological observation showed that circulating PGCs (cPGCs) were present in the blood as large, round, individually dispersed cells, whereas gonadal PGCs (gPGCs) had migrated out of the vasculature and colonized the gonads, where they exhibited a typical clustered distribution (Figure 1C). Sex was determined for each embryo by PCR amplification of the *CHD1* gene using extracted genomic DNA.

Immunofluorescence staining showed that all four groups of cells, including cPGC_M, cPGC_F, gPGC_M, and gPGC_F, exhibited strong SSEA-1-positive fluorescence signals. These signals were spatially consistent with DiI labeling and DAPI nuclear staining in the merged images, indicating that the isolated cells possessed PGC-associated phenotypic characteristics (Figure 1D). Collectively, these results demonstrate that the isolated cells displayed typical morphological and immunophenotypic features of PGCs and that the sex of each sample was reliably identified, supporting their suitability for subsequent RNA-seq analysis. The examined culture fields were visually dominated by cells with PGC-like morphology; however, this observation was qualitative, and neither cell population purity nor contamination by non-PGC cell types was quantitatively determined.

### 3.2. Quality Control of RNA-Seq Data and Inter-Sample Clustering Analysis

After quality filtering of the raw RNA-seq data, 35.74–47.83 million clean reads were obtained for each sample, corresponding to 5.37–7.19 Gb of clean bases. The proportion of clean reads ranged from 98.39% to 98.85%. The GC content of all samples ranged from 45.47% to 46.15%, and the Q20 and Q30 values were no lower than 98.96% and 95.93%, respectively. These results indicate that the sequencing data were of high quality and suitable for subsequent differential expression analysis and functional enrichment analysis (Table 1).

To evaluate the overall quality of the RNA-seq data and the consistency among samples, principal component analysis, sample correlation analysis, and expression distribution analysis were performed for all samples. The PCA results showed that PGC samples from different sources and sexes exhibited a certain degree of separation at the global transcriptomic level. Biological replicates within each group showed generally consistent distributions, and no obvious outlier samples were observed (Figure 1E). In addition, the density curves of log10(FPKM) expression values were largely consistent across all samples, suggesting balanced global expression distributions among different samples (Figure 1F). Nevertheless, the replicate points were not identical and showed visible within-group dispersion in the PCA, reflecting embryo-to-embryo variability. With three biological replicates per group, this variability can only be described qualitatively and cannot be estimated precisely or generalized to the broader Silkie chicken population.

### 3.3. Transcriptomic Dynamics of PGCs from the Circulating Stage to the Gonadal Colonization Stage

**Figure 2:**
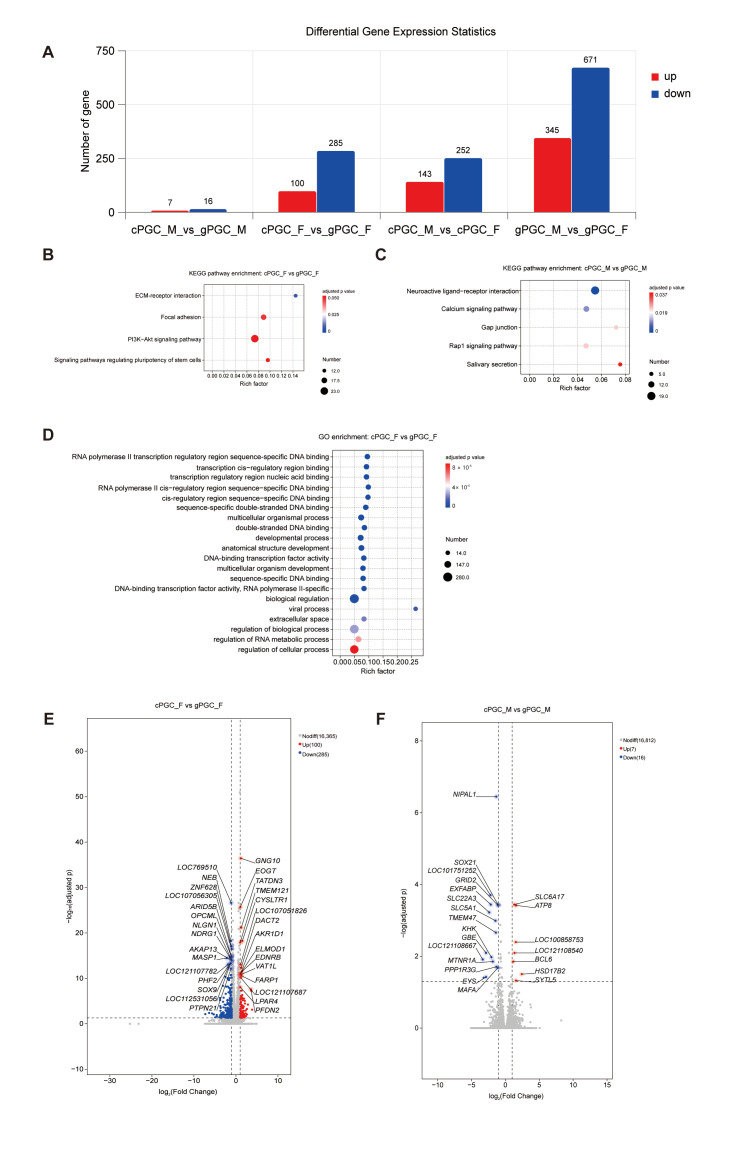
**Transcriptomic dynamics of chicken primordial germ cells (PGCs) during the blood-to-gonad transition.** (**A**) Numbers of differentially expressed genes (DEGs; adjusted *p* < 0.05 and |log2(Fold Change)| ≥ 1) identified in four pairwise comparisons: cPGC_M versus gPGC_M, cPGC_F versus gPGC_F, cPGC_M versus cPGC_F, and gPGC_M versus gPGC_F. Red bars indicate upregulated genes, whereas blue bars indicate downregulated genes. (**B**) KEGG pathway enrichment analysis of DEGs between cPGC_F and gPGC_F (adjusted *p* < 0.05). Dot size represents the number of DEGs assigned to each pathway, and dot color represents the adjusted *p* value. (**C**) KEGG pathway enrichment analysis of DEGs between cPGC_M and gPGC_M. Dot size represents the number of genes, and dot color represents the adjusted *p* value. (**D**) Gene Ontology (GO) enrichment analysis of DEGs between cPGC_F and gPGC_F. Dot size represents the number of genes, and dot color represents the adjusted *p* value. (**E**) Volcano plot of DEGs between cPGC_F and gPGC_F. Red dots represent upregulated genes (*n* = 100), blue dots represent downregulated genes (*n* = 285), and gray dots represent non-differentially expressed genes (*n* = 16,365). Representative genes are labeled. (**F**) Volcano plot of DEGs between cPGC_M and gPGC_M. Red dots represent upregulated genes (*n* = 7), blue dots represent downregulated genes (*n* = 16), and gray dots represent non-differentially expressed genes (*n* = 16,812). Representative genes are labeled. For all comparisons, the pair is written as A versus B, whereas log2(Fold Change) was calculated as B/A; therefore, red/upregulated genes have higher expression in the latter group, and blue/downregulated genes have higher expression in the former group.

To characterize the transcriptomic changes associated with the transition of PGCs from the circulating stage in embryonic blood (cPGCs) to the gonadal colonization stage (gPGCs), differentially expressed genes (DEGs) were identified between the two developmental stages within the same sex. Using the criteria of adjusted *p* < 0.05 and |log2(Fold Change)| ≥ 1, a total of 385 DEGs were identified in female PGCs between cPGC_F and gPGC_F, including 100 upregulated and 285 downregulated genes in gPGC_F relative to cPGC_F. In contrast, only 23 DEGs were detected in male PGCs between cPGC_M and gPGC_M, including 7 upregulated and 16 downregulated genes in gPGC_M relative to cPGC_M (Figure 2A and Figure S1). Within this dataset, more DEGs were detected in the female than in the male stage comparison. However, with three replicates per group, this difference should not be interpreted as definitive evidence that female PGCs universally undergo greater or earlier developmental remodeling.

KEGG enrichment analysis showed that DEGs in the female stage comparison were significantly enriched (adjusted *p* < 0.05) in pathways including ECM–receptor interaction, focal adhesion, PI3K–Akt signaling pathway, and signaling pathways regulating pluripotency of stem cells (Figure 2B). These pathways have previously been linked to cell–matrix interaction, cell adhesion, proliferation/survival, and stem cell fate in PGC biology [23,24]; however, enrichment in the present study denotes association and does not demonstrate causal regulation of migration or gonadal colonization. GSEA further revealed significant enrichment of the ECM–receptor interaction gene set in cPGC_F (adjusted *p* < 0.05; Figure S2A). Although the focal adhesion and PI3K–Akt signaling pathways showed similar enrichment trends toward cPGC_F, neither met the adjusted *p* < 0.05 threshold (Figure S2B,C).

GO enrichment analysis further showed that DEGs (adjusted *p* < 0.05) in the female stage comparison were enriched in terms related to regulation of transcription by RNA polymerase II, DNA-binding transcription factor activity, and multicellular organism development (Figure 2D). At the gene level, genes upregulated in gPGC_F included *EDNRB*, *LPAR4*, *FARP1*, *DACT2*, and *AKR1D1*, whereas genes that were expressed at relatively high levels in cPGC_F and downregulated after gonadal colonization included *SOX2*, *SOX9*, *NDRG1*, *PHF2*, and *ARID5B* (Figure 2C). The lower expression of *SOX2* in gPGC_F is compatible with a change in pluripotency-associated transcriptional status, but expression data alone cannot establish a functional loss of pluripotency or the initiation of differentiation [25].

### 3.4. Sex-Biased Transcriptomic Differences in PGCs and Their Expansion During Development

To investigate sex-biased transcriptomic differences in PGCs and their changes during development, we compared differentially expressed genes (DEGs) between male and female PGCs at both the circulating stage (cPGC_M vs. cPGC_F) and the gonadal colonization stage (gPGC_M vs. gPGC_F). Using the criteria of adjusted *p* < 0.05 and |log2(Fold Change)| ≥ 1, a total of 395 sex-biased DEGs were identified at E2.5, including 143 genes highly expressed in cPGC_F and 252 genes highly expressed in cPGC_M. By E8.5, the number of sex-biased DEGs increased to 1016, including 345 genes highly expressed in gPGC_F and 671 genes highly expressed in gPGC_M, representing an approximately 2.6-fold increase relative to E2.5 (Figure 2A). This increase describes the number of detected DEGs in the present sample set and does not, by itself, demonstrate a mechanism of sex differentiation. The overlap among the DEG sets identified in the four pairwise comparisons is summarized in Figure S1.

**Figure 3:**
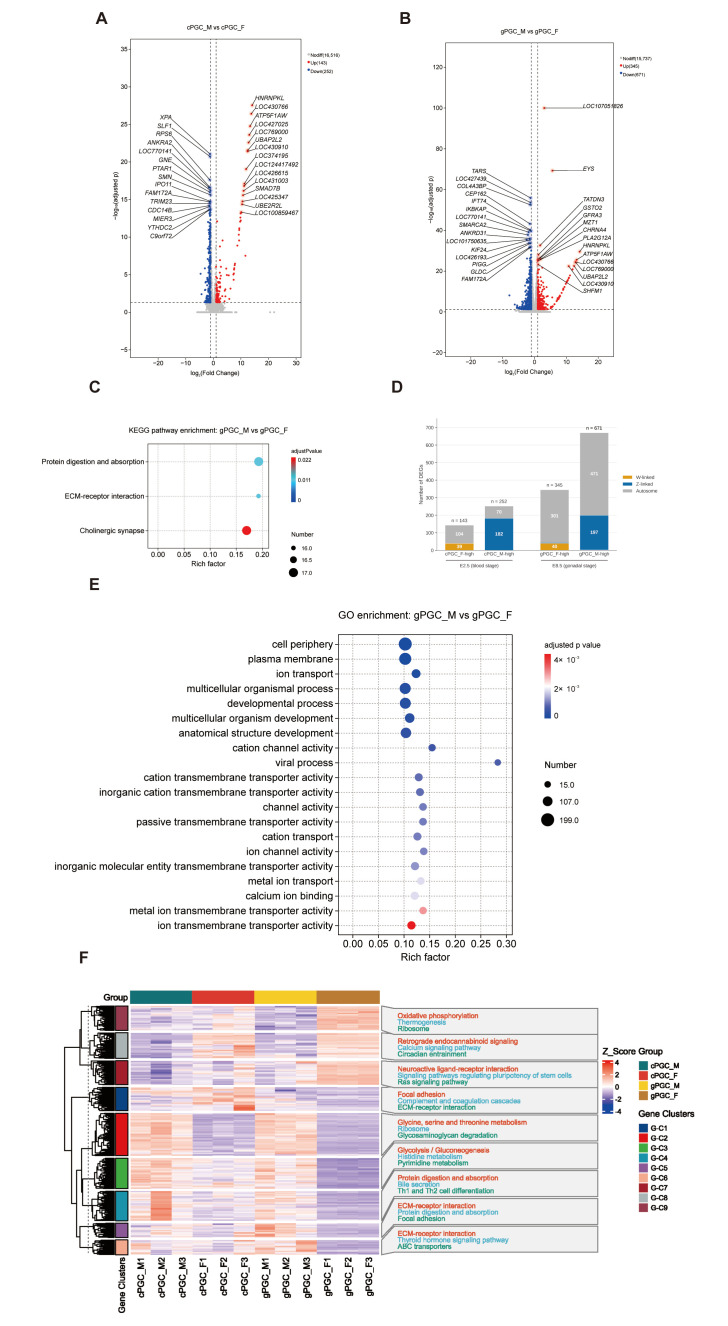
**Expansion of sex-biased transcriptomic differences in chicken PGCs from the blood-circulating stage to the gonad-colonized stage.** (**A**,**B**) Volcano plots of differentially expressed genes (DEGs; adjusted *p* < 0.05 and |log2(Fold Change)| ≥ 1) between (**A**) cPGC_M and cPGC_F and (**B**) gPGC_M and gPGC_F. In (**A**), 143 genes were expressed at higher levels in cPGC_F, 252 genes were expressed at higher levels in cPGC_M, and 16,516 genes were classified as non-DEGs. In (**B**), 345 genes were expressed at higher levels in gPGC_F, 671 genes were expressed at higher levels in gPGC_M, and 15,737 genes were classified as non-DEGs. Representative genes are labeled. (**C**) KEGG pathway enrichment analysis of DEGs between gPGC_M and gPGC_F (adjusted *p* < 0.05). Dot size represents the number of genes, and dot color represents the adjusted *p* value. (**D**) Chromosomal distribution of sex-biased DEGs at the two developmental stages. Bars are stacked according to chromosomal origin: W-linked (orange), Z-linked (blue), and autosomal (gray). At E2.5, cPGC_F-high genes included 39 W-linked and 104 autosomal genes (*n* = 143), whereas cPGC_M-high genes included 182 Z-linked and 70 autosomal genes (*n* = 252). At E8.5, gPGC_F-high genes included 40 W-linked, 4 Z-linked, and 301 autosomal genes (*n* = 345), whereas gPGC_M-high genes included 3 W-linked, 197 Z-linked, and 471 autosomal genes (*n* = 671). The number of sex chromosome-linked DEGs increased slightly from 221 at E2.5 to 244 at E8.5, whereas the number of autosomal DEGs increased from 174 to 772, representing an approximately 4.4-fold expansion. (**E**) Gene Ontology (GO) enrichment analysis of DEGs between gPGC_M and gPGC_F. Dot size represents the number of genes, and dot color represents the adjusted *p* value. (**F**) Hierarchical clustering heatmap of all DEGs across the 12 samples, with three biological replicates per group. DEGs were partitioned into nine expression pattern clusters (G-C1–G-C9), and the top three KEGG pathways enriched in each cluster are annotated on the right. Colors indicate Z-score-normalized expression levels. For both volcano plots, the pair is written as male group A versus female group B, whereas log2(Fold Change) was calculated as B/A; positive values on the right indicate higher expression in the female group, and negative values on the left indicate higher expression in the male group.

At E2.5, sex-biased DEGs were not significantly enriched in any KEGG pathway at adjusted *p* < 0.05, and GO enrichment analysis identified only one significant term, “viral process”. The volcano plot showed that genes highly expressed in cPGC_F were mainly represented by W chromosome-linked genes, including *HNRNPKL*, *ATP5F1AW*, *UBAP2L2*, *SMAD7B*, and *UBE2R2L*. In contrast, genes highly expressed in cPGC_M were mainly represented by Z chromosome-linked genes, including *XPA*, *RPS6*, *ANKRA2*, *SMN*, *IPO11*, *FAM172A*, *TRIM23*, *CDC14B*, *YTHDC2*, and *C9orf72* (Figure 3A). GSEA revealed significant enrichment of the ribosome gene set toward cPGC_M (adjusted *p* < 0.05), whereas the TGF-β signaling pathway did not meet the adjusted *p* < 0.05 threshold. Thus, although *SMAD7*/*SMAD7B* showed clear sex-biased expression at the individual gene level, the overall pathway-level difference in TGF-β signaling was limited at this stage (Figure S3C,D).

At E8.5, sex-biased DEGs were significantly enriched (adjusted *p* < 0.05) in three KEGG pathways: protein digestion and absorption, ECM–receptor interaction, and cholinergic synapse (Figure 3C). GSEA further showed that the protein digestion and absorption pathway was significantly enriched toward gPGC_M (adjusted *p* < 0.05), whereas ECM–receptor interaction showed a male-biased trend toward gPGC_M but did not meet the adjusted *p* < 0.05 threshold (Figure S3A,B). GO enrichment analysis indicated that sex-biased DEGs at E8.5 were mainly associated with cell periphery, plasma membrane, and ion-channel- or transmembrane-transporter activity-related terms (adjusted *p* < 0.05; Figure 3E). The volcano plot showed that genes highly expressed in gPGC_F included W-linked markers such as *HNRNPKL*, *ATP5F1AW*, and *UBAP2L2*, as well as autosomal genes including *EYS*, *TATDN3*, *GSTO2*, *CHRNA4*, *MZT1*, *PLA2G12A*, *SHFM1*, and *GFRA3*. Genes highly expressed in gPGC_M included *TARS*, *COL4A3BP*, *CEP162*, *IFT74*, *SMARCA2*, *ANKRD31*, *KIF24*, *PIGG*, and *GLDC* (Figure 3B).

The female-biased expression of W-linked genes and male-biased expression of Z-linked genes were consistent with female-specific W-linked expression in ZW individuals [26] and gene-specific dosage differences among avian Z-linked genes [27].The male sex-determining gene *DMRT1*, located on the Z chromosome, showed male-biased expression at both developmental stages, with stronger statistical support at E8.5 [28]. In contrast, the W-linked gene *HINTW* did not meet the adjusted *p* < 0.05 threshold at E2.5 but was significantly more highly expressed in gPGC_F at E8.5.

To further clarify the chromosomal basis underlying the expansion of sex-biased transcriptomic differences, we analyzed the chromosomal distribution of sex-biased DEGs at the two developmental stages (Figure 3D). W-linked DEGs were predominantly enriched among female-biased genes, with 39 genes highly expressed in cPGC_F and 40 genes highly expressed in gPGC_F. In contrast, Z-linked DEGs were mainly enriched among male-biased genes, with 182 genes highly expressed in cPGC_M and 197 genes highly expressed in gPGC_M. Overall, the number of sex chromosome-linked DEGs increased only slightly from 221 at E2.5 to 243 at E8.5. By comparison, the number of autosomal DEGs increased from 174 to 772, representing an approximately 4.4-fold expansion. Within this dataset, the numerical expansion of sex-biased DEGs was therefore dominated by autosomal genes rather than by a large increase in sex chromosome-linked DEGs. This distribution is descriptive and does not establish that autosomal expression changes causally drive PGC sex differentiation.

To obtain an integrated view of global expression patterns among the four PGC groups, all DEGs were subjected to Z-score normalization and hierarchical clustering, resulting in nine expression pattern clusters, designated G-C1 to G-C9 (Figure 3F). These clusters showed distinct stage- or sex-biased expression patterns and were enriched in pathways including oxidative phosphorylation, ribosome, ECM–receptor interaction, focal adhesion, neuroactive ligand–receptor interaction, Ras signaling pathway, and calcium signaling pathway. The clusters describe coordinated expression patterns in the present dataset; their biological functions and regulatory roles remain to be tested experimentally.

### 3.5. Targeted RT-qPCR Corroboration of Six Female Stage-Associated Genes

**Figure 4:**
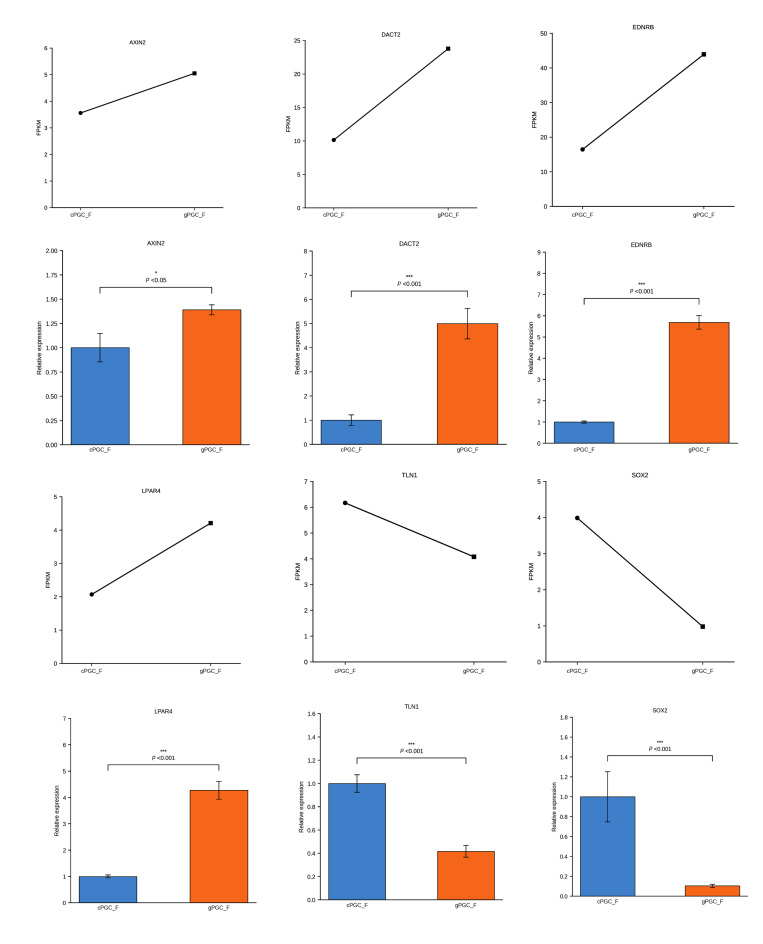
Targeted RT-qPCR corroboration of six genes associated with the developmental transition of female chicken primordial germ cells (PGCs). Line plots show the FPKM values obtained by RNA-seq for cPGC_F and gPGC_F, whereas bar plots show relative gene expression determined by RT-qPCR. *AXIN2*, *DACT2*, *EDNRB*, and *LPAR4* were upregulated in gPGC_F, whereas *TLN1* and *SOX2* were downregulated. Relative expression was calculated using the 2^−ΔΔCt^ method, with cPGC_F set to 1. Error bars represent the standard error of the mean (SEM) of three biological replicates (*n* = 3). Statistical differences between groups were evaluated using a two-tailed unpaired Student’s *t*-test. * *p* < 0.05; *** *p* < 0.001. cPGC_F, female circulating primordial germ cells; gPGC_F, female gonad-derived primordial germ cells; FPKM, fragments per kilobase of transcript per million mapped reads; RT-qPCR, quantitative real-time PCR. The assay included female samples only and should not be interpreted as validation of the male or sex-biased RNA-seq comparisons (the original RT-qPCR pictures can be found in File S4).

To provide targeted corroboration of selected RNA-seq trends, six genes associated with cell adhesion, migration, signaling, or pluripotency in the literature were examined by RT-qPCR in female PGCs. *AXIN2*, *DACT2*, *EDNRB*, and *LPAR4* were expressed at significantly higher levels in gPGC_F than in cPGC_F, whereas *TLN1* and *SOX2* were significantly downregulated after gonadal colonization (*p* < 0.05; Figure 4). The direction of change was consistent with the corresponding RNA-seq-derived FPKM trends for these six genes. This limited analysis supports only the selected female stage-associated expression trends; it does not independently validate the male stage comparison, the sex-biased comparisons, or any functional role of these genes.

## 4. Discussion

The female developmental-stage comparison identified enrichment of genes annotated to ECM–receptor interaction, focal adhesion, PI3K–Akt signaling pathway, and signaling pathways regulating pluripotency of stem cells. These enrichments show that expression differences between cPGC_F and gPGC_F overlap with functional categories related to cell–matrix interaction, adhesion, proliferation/survival, and stem cell state; they do not demonstrate that these pathways mechanistically control migration or gonadal colonization in the present study. GSEA showed significant enrichment of the ECM–receptor interaction gene set in cPGC_F, whereas focal adhesion and PI3K–Akt signaling showed similar directional tendencies but did not meet the adjusted *p* < 0.05 threshold. Previous studies have implicated extracellular–matrix interactions and PI3K–Akt-related signaling in avian PGC biology [12,23,24], providing biological context for the observed associations. At the gene level, *EDNRB*, *LPAR4*, *FARP1*, and *DACT2* differed between cPGC_F and gPGC_F; these genes should be regarded as candidate markers or hypotheses for future functional testing rather than as confirmed regulators of migration or signaling. Similarly, the lower expression of *SOX2*, *SOX9*, *PHF2*, and *ARID5B* in gPGC_F is compatible with an altered pluripotency-associated expression state but does not establish the initiation of differentiation [25].

In contrast, only 23 differentially expressed genes were identified in male PGCs within the same developmental window, indicating a smaller number of detected expression differences than in the female comparison. The enriched pathways in male PGCs were mainly associated with cell communication-related processes, including neuroactive ligand–receptor interaction, calcium signaling pathway, gap junction, and Rap1 signaling pathway. Female PGCs begin oogonial differentiation at approximately E8.0, whereas male PGCs usually initiate prospermatogonial differentiation after E13.0 and remain largely undifferentiated at E8.5 [6]. This known difference in developmental timing offers one possible explanation for the observed transcriptomic pattern. However, the present data cannot establish that gPGC_F had entered a differentiation program or that gPGC_M was transcriptionally stable, because the smaller male DEG count may also reflect inter-individual variability and limited statistical power.

From the perspective of sex-biased transcriptomic divergence, this study found that the number of sex-biased genes increased from 395 at E2.5 to 1016 at E8.5. Chromosomal distribution analysis showed that the number of sex chromosome-linked sex-biased genes increased only slightly, from 221 to 243, whereas the number of autosomal sex-biased genes increased from 174 to 772. Thus, the numerical increase in detected sex-biased DEGs was dominated by autosomal genes in this dataset; this is an association and does not identify a causal regulatory hierarchy for sex differentiation. At E2.5, sex-biased differences were primarily characterized by sex chromosome-linked genes. Genes highly expressed in cPGC_F were mostly W-linked genes, including *HNRNPKL*, *ATP5F1AW*, *UBAP2L2*, *SMAD7B*, and *UBE2R2L*, consistent with the female-specific expression pattern of W-linked genes in ZW individuals [26]. In contrast, genes highly expressed in cPGC_M were predominantly located on the Z chromosome, which may reflect gene-specific dosage differences among non-compensated or partially compensated avian Z-linked genes [27]. Although *SMAD7*/*SMAD7B* showed stable sex-biased expression at the individual gene level, the TGF-β signaling pathway did not meet the adjusted *p* < 0.05 threshold in GSEA, suggesting that this difference may primarily reflect W/Z homolog-associated gene-level divergence rather than a coordinated pathway-level alteration.

By E8.5, sex-biased differences further expanded to autosomal genes and functional pathways, with significant enrichment (adjusted *p* < 0.05) in protein digestion and absorption, ECM–receptor interaction, and cholinergic synapse. The Z-linked male sex-determining gene *DMRT1* was more highly expressed in males at both developmental stages, with stronger statistical support at E8.5, consistent with its established role in avian male sex determination [28]. The W-linked gene *HINTW* was not significantly differentially expressed at E2.5 but was significantly upregulated in gPGC_F at E8.5, indicating stage-associated expression. Recent functional studies suggest that *HINTW* may contribute to female differentiation through hormone-related and UBE2I-associated mechanisms [29,30]; however, its role in PGCs specifically remains unverified. In addition, autosomal genes such as *GFRA3* showed pronounced sex-biased expression at E8.5, whereas typical gonadal somatic sex-determination genes, including *FOXL2*, *CYP19A1*, and *AMH*, did not meet the adjusted *p* < 0.05 threshold in PGCs. *GFRA3* and the other autosomal DEGs should therefore be regarded as sex-associated candidate markers rather than verified regulators of PGC sex differentiation. The observed expression pattern is consistent with PGCs and gonadal somatic cells having distinct sex-associated transcriptional features, but this interpretation requires validation in larger cohorts and functional models [31].

These observations are broadly consistent with recent transcriptomic studies. Huang et al. (2022) and Rengaraj et al. (2022) identified adhesion-, proliferation-, and migration-associated expression programs in circulating or actively migrating chicken PGCs [24,32]. Doddamani et al. (2023) reported intrinsic sex-biased transcription in migratory chicken PGCs [26], whereas Ichikawa et al. (2022) identified stage- and sex-associated regulatory signatures in avian PGCs [31]. The present dataset directly compares both sexes at E2.5 and E8.5 and therefore complements these studies, but the two-time-point design cannot reconstruct continuous transcriptional trajectories. Recent single-cell analysis of gonadal PGCs in zebra finch also illustrates the cellular heterogeneity that bulk RNA-seq cannot resolve [33].

## Figures and Tables

**Table 1:** Summary of RNA-seq data quality after filtering.

Sample	Raw Reads (M)	Clean Reads (M)	Clean Bases (Gb)	Clean Reads (%)	GC Content (%)	Q20 (%)	Q30 (%)
cPGC_M1	41.60	41.01	6.18	98.59	45.91	98.99	95.93
cPGC_M2	41.62	41.10	6.19	98.75	45.58	99.09	96.39
cPGC_M3	40.83	40.17	6.05	98.39	45.77	98.96	96.01
cPGC_F1	41.17	40.66	6.12	98.78	45.71	99.09	96.33
cPGC_F2	46.54	45.94	6.87	98.70	46.04	99.02	96.36
cPGC_F3	46.44	45.91	6.91	98.85	45.99	99.12	96.39
gPGC_M1	39.03	38.53	5.79	98.72	45.81	99.02	96.13
gPGC_M2	48.44	47.83	7.19	98.74	45.74	99.00	95.97
gPGC_M3	39.11	38.58	5.79	98.64	46.15	99.01	96.24
gPGC_F1	43.86	43.31	6.51	98.76	45.61	99.02	96.09
gPGC_F2	36.66	36.19	5.44	98.72	45.47	99.07	96.38
gPGC_F3	36.18	35.74	5.37	98.78	45.63	99.05	96.29

Note: cPGC indicates blood-derived circulating primordial germ cells, whereas gPGC indicates gonad-derived primordial germ cells. F and M indicate female and male samples, respectively. Raw reads and clean reads are expressed in millions of reads, and clean bases are expressed in gigabases. Clean reads (%) represents the proportion of reads retained after quality filtering. Q20 and Q30 indicate the percentages of bases with sequencing quality scores higher than 20 and 30, respectively. Unit abbreviations are as follows: M denotes million reads, Gb denotes gigabases, and % denotes percentage.

## Data Availability

The raw RNA-sequencing data generated in this study are openly available in NCBI Sequence Read Archive (SRA) under BioProject accession number PRJNA1481453.

## Supplementary Materials

The following supporting information can be downloaded at: https://www.mdpi.com/article/10.3390/vetsci13070662/s1, Supplementary Methods: Immunofluorescence identification of PGCs and RT-qPCR validation; Table S1: Primer sequences used for RT-qPCR validation; Figure S1: Overlap of differentially expressed genes among the four pairwise comparisons; Figure S2: Gene set enrichment analysis of developmental-stage-associated pathways in chicken primordial germ cells; Figure S3: Gene set enrichment analysis of sex-biased pathways in chicken primordial germ cells; File S4: Original images of RT-qPCR.

## Abbreviations

The following abbreviations are used in this manuscript:

PGC	Primordial germ cell
cPGC	Circulating primordial germ cell
gPGC	Gonad-derived primordial germ cell
HH	Hamburger–Hamilton
RNA-seq	RNA sequencing
RT-qPCR	Reverse transcription quantitative real-time PCR
DEG	Differentially expressed gene
PCA	Principal component analysis
FPKM	Fragments per kilobase of transcript per million mapped reads
GO	Gene Ontology
KEGG	Kyoto Encyclopedia of Genes and Genomes
GSEA	Gene set enrichment analysis
ECM	Extracellular matrix
RIN	RNA integrity number
SEM	Standard error of the mean
