# Supplementary material for: Transcriptional Profiling of Primordial Germ Cells During Chicken Embryonic Development
Source: Vet Sci. 2026 Jul 7;13(7):662. doi: 10.3390/vetsci13070662 (PMC13418713; doi:10.3390/vetsci13070662)
Supplement: Supplementary file 1 [file vetsci-13-00662-s001.zip › Supplementary_Materials.pdf]

Supplementary Materials

Transcriptional Profiling of Primordial Germ Cells during Chicken Embryonic Development

Mingyang Jin, Jingkang Huang, Chao Qin, Kaixuan Yang, Fuquan Xiao and He Meng

This file contains Supplementary Methods, Table S1, and Figures S1–S3.

Supplementary Methods

S1. Immunofluorescence Identification of PGCs

Cultured primordial germ cells (PGCs) were evaluated by morphological observation and indirect immunofluorescence staining for stage-specific embryonic antigen-1 (SSEA-1). A mouse anti-SSEA-1 antibody (clone MC-480; Developmental Studies Hybridoma Bank, Iowa City, IA, USA) was used for immunostaining. Cells were additionally labeled with DiI, and nuclei were counterstained with DAPI. SSEA-1, DiI, and DAPI fluorescence signals were acquired in the corresponding channels and merged to assess their spatial colocalization.

S2. RT-qPCR Validation

Total RNA was extracted from PGC samples using the FastPure Cell/Tissue Total RNA Isolation Kit V2 (RC112-01; Vazyme, Nanjing, China). First-strand cDNA was synthesized using the HiScript III 1st Strand cDNA Synthesis Kit +gDNA wiper (R312-02; Vazyme) according to the manufacturer's instructions. RT-qPCR was performed using ChamQ Blue Universal SYBR qPCR Master Mix (Q312-02; Vazyme) on a 7500 Real-Time PCR System (Applied Biosystems, Thermo Fisher Scientific). *GAPDH* was used as the reference gene. Relative expression was calculated using the $2^{-\Delta\Delta Ct}$ method, with cPGC-F as the calibrator. Three independent biological replicates were analyzed for each group. The primer sequences are listed in Table S1.

Table S1. Primer sequences used for RT-qPCR validation.

Gene	Forward primer (5′–3′)	Reverse primer (5′–3′)
AXIN2	CCTTAGACTTCTGGTTTGCC	TCTGTGCCTGATCAAACATG
DACT2	GCGAGGCTCAGTGTGTTTG	GGGGCTTGGGTAAGGGTAC
EDNRB	GGAGGAAGACCATTGAGGCA	ATTCCGTGACTTTGGGTGCT
SOX2	GGAGGCTATGGGATGATGC	ACGAGGAGGTGACTACGGG
TLN1	CGGATGAAGACCCAAAGAAG	GGGCACATATAGTCATCAGC
LPAR4	ACTCTTCTTGTATGCTCTC	TTTATCTGGGTCTTTATGT
GAPDH	CCTCTCTGGCAAAGTCCAAG	CATCTGCCCATTTGATGTTG

Note: All primer sequences are presented in the 5′–3′ orientation. *GAPDH* was used as the reference gene.

Supplementary Figures

S1

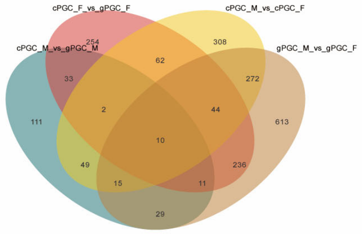

Figure S1. Overlap of differentially expressed genes (DEGs) among the four pairwise comparisons. The Venn diagram shows the numbers of unique and shared DEGs identified in cPGC_M vs. gPGC_M, cPGC_F vs. gPGC_F, cPGC_M vs. cPGC_F, and gPGC_M vs. gPGC_F using adjusted P < 0.05 and $|\log_2FC| \geq 1$.

S2

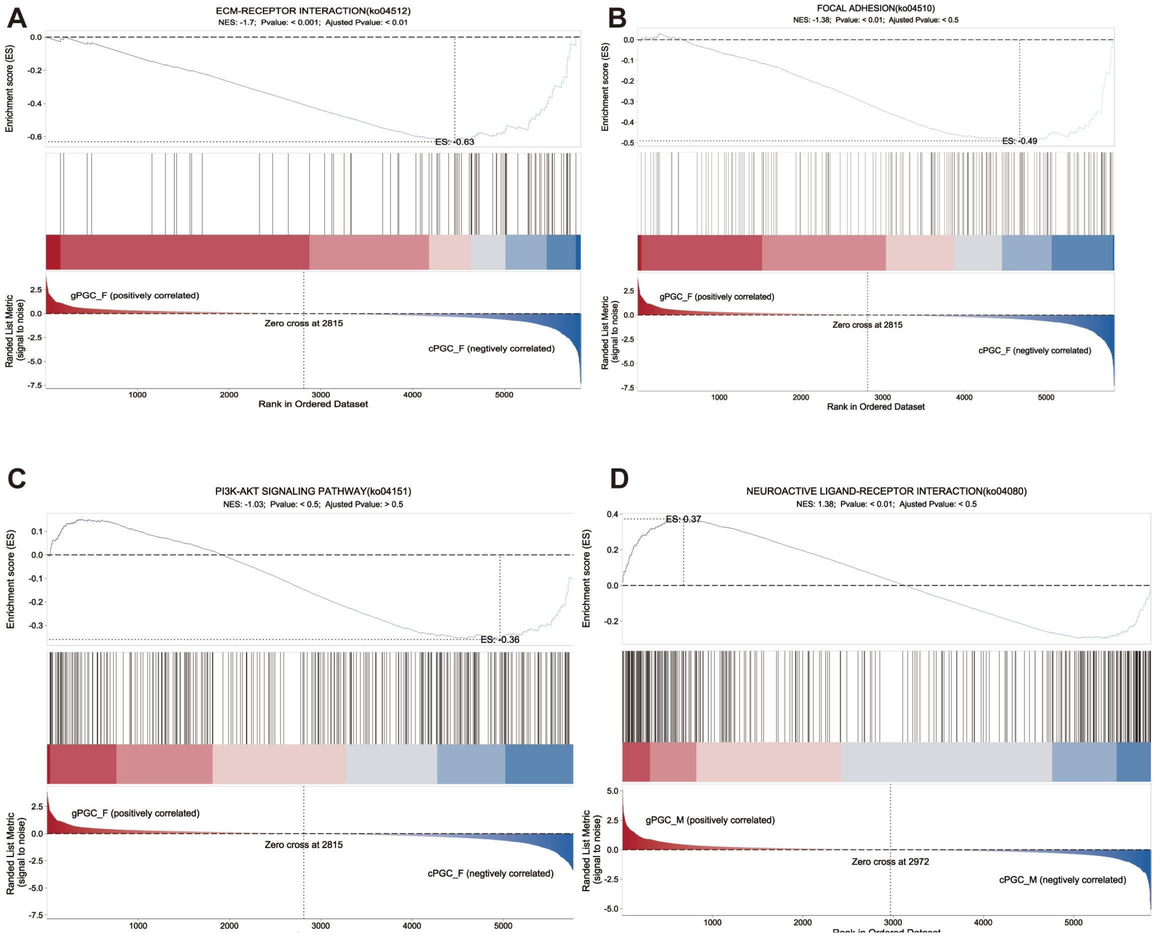

Figure S2. Gene set enrichment analysis (GSEA) of developmental-stage-associated pathways in chicken primordial germ cells (PGCs). (A–C) GSEA plots for the cPGC_F vs. gPGC_F comparison: (A) ECM–receptor interaction (ko04512), (B) Focal adhesion (ko04510), and (C) PI3K–Akt signaling pathway (ko04151). Negative normalized

enrichment scores (NESs) indicate enrichment toward cPGC_F; ECM–receptor interaction reached statistical significance, whereas Focal adhesion and PI3K–Akt signaling showed non-significant trends. (D) GSEA plot for Neuroactive ligand–receptor interaction (ko04080) in the cPGC_M vs. gPGC_M comparison. A positive NES indicates enrichment toward gPGC_M.

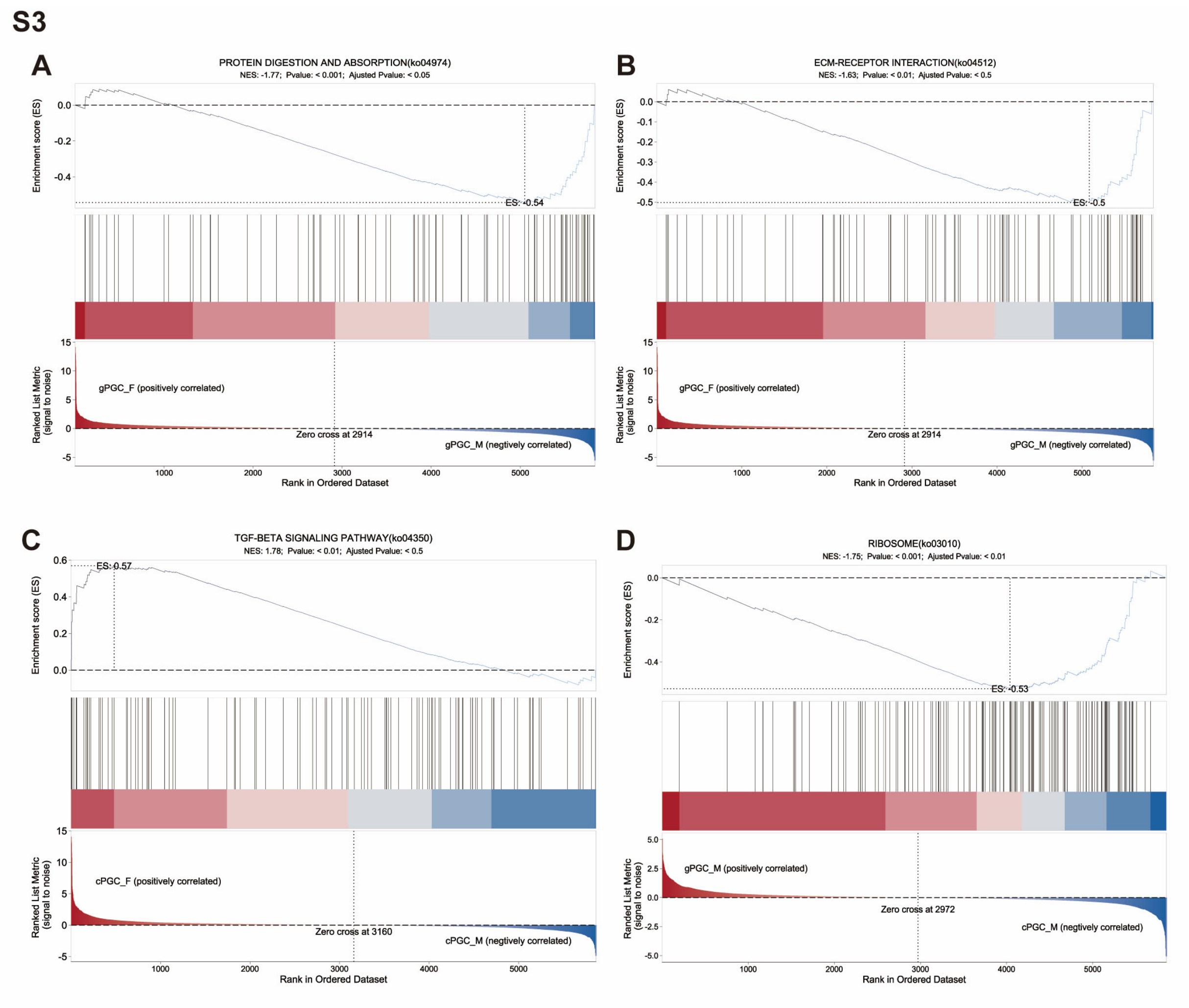

Figure S3. Gene set enrichment analysis (GSEA) of sex-biased pathways in chicken primordial germ cells (PGCs). (A,B) GSEA plots for the gPGC_M vs. gPGC_F comparison: (A) Protein digestion and absorption (ko04974) and (B) ECM–receptor interaction (ko04512). Negative normalized enrichment scores (NESs) indicate enrichment toward gPGC_M; Protein digestion and absorption reached statistical significance, whereas ECM–receptor interaction showed a non-significant trend. (C,D) GSEA plots for the cPGC_M vs. cPGC_F comparison: (C) TGF-β signaling pathway (ko04350) and (D) Ribosome (ko03010). The positive NES for TGF-β signaling indicates a trend toward cPGC_F, whereas the negative NES for Ribosome indicates enrichment toward cPGC_M. Ribosome reached statistical significance, whereas TGF-β signaling was not statistically significant.
