# Supplementary figures and images for: Transcriptional Profiling of Primordial Germ Cells During Chicken Embryonic Development
Source: Vet Sci. 2026 Jul 7;13(7):662. doi: 10.3390/vetsci13070662 (PMC13418713; doi:10.3390/vetsci13070662)

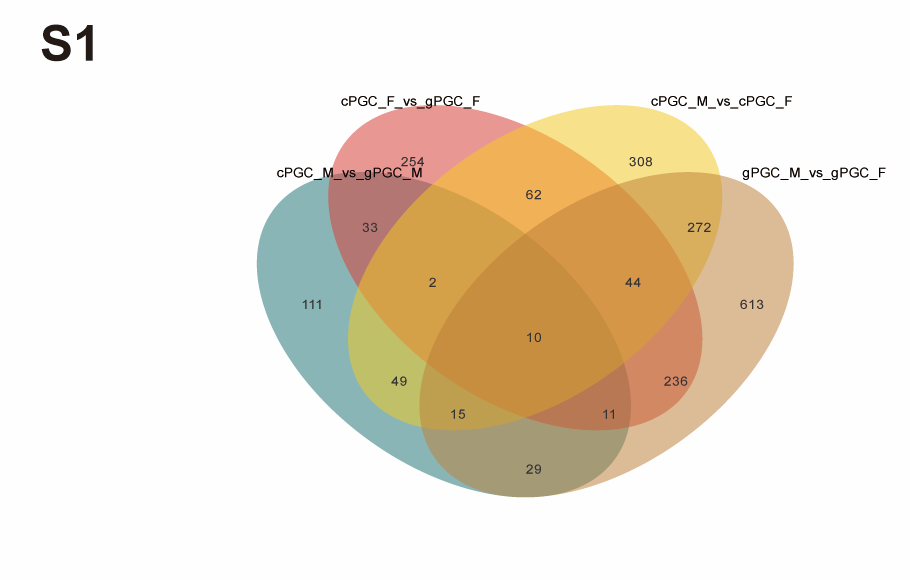

Supplement: Supplementary file 1 [file vetsci-13-00662-s001.zip › S1_300dpi.tiff]

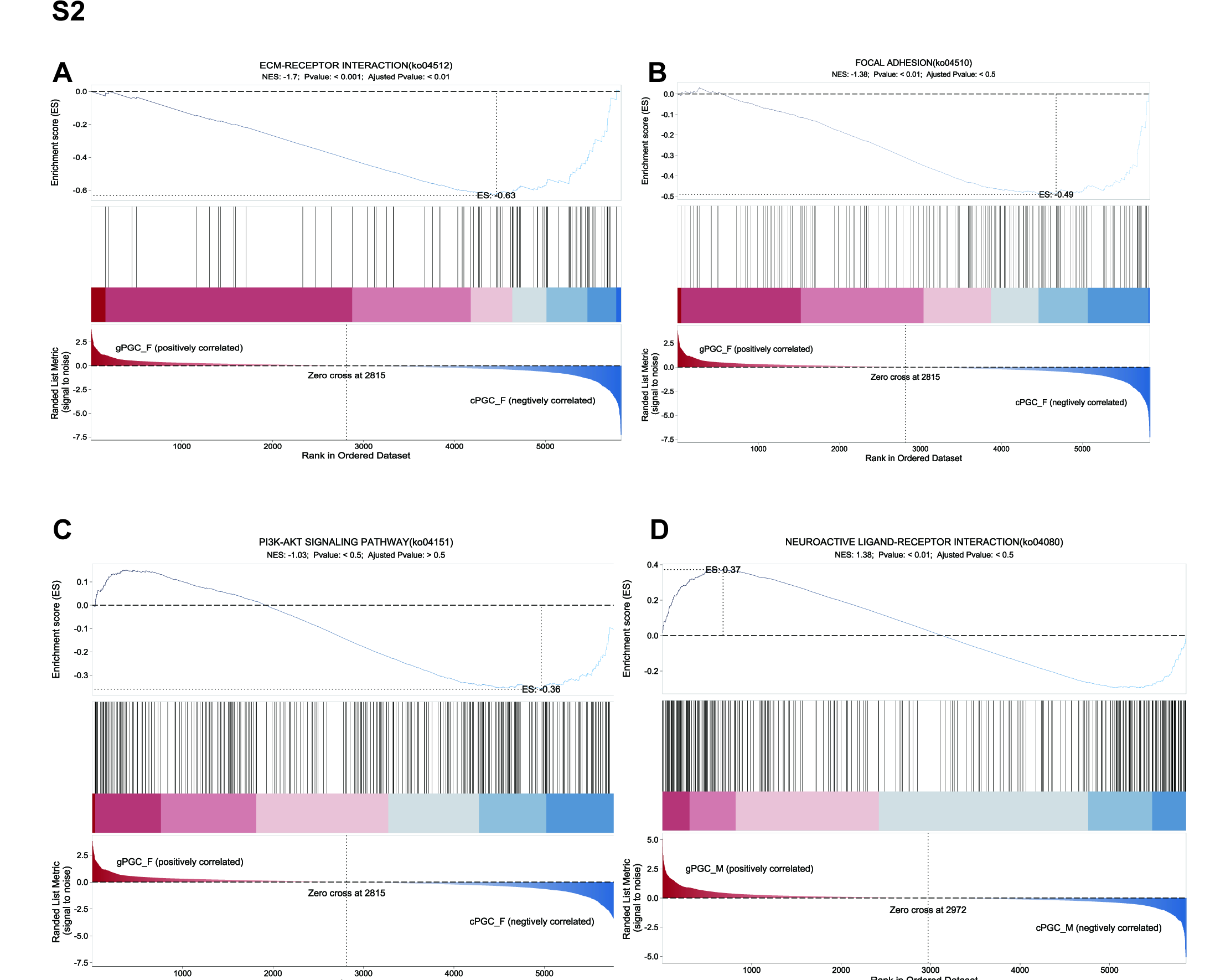

Supplement: Supplementary file 1 [file vetsci-13-00662-s001.zip › S2_300dpi.tiff]

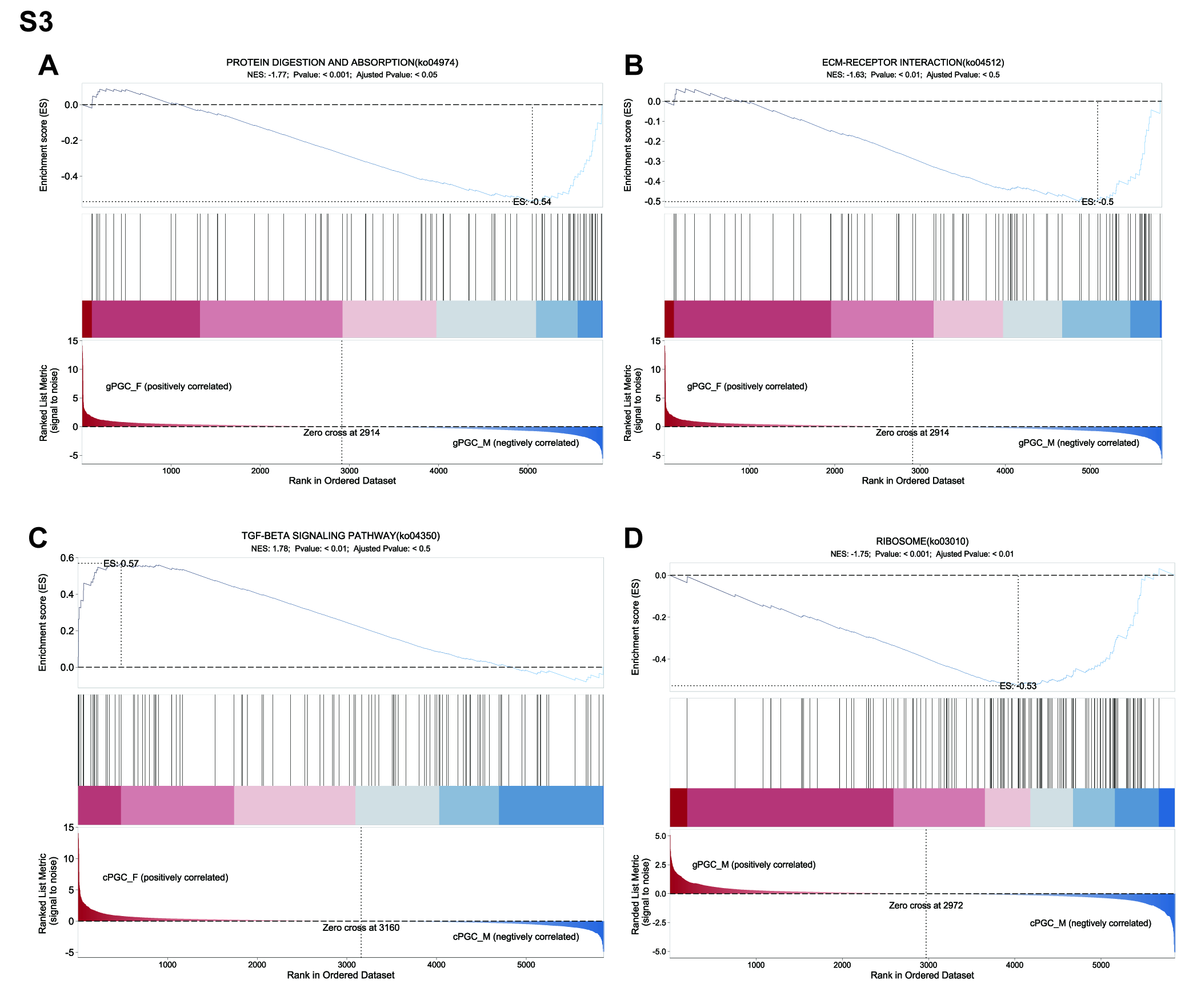

Supplement: Supplementary file 1 [file vetsci-13-00662-s001.zip › S3_300dpi.tif]
